# Label-free quantitative proteomics identifies transforming growth factor β1 (TGF-β1) as an inhibitor of adipogenic transformation in OP9-DL1 cells and primary thymic stromal cells

**DOI:** 10.1186/s13578-019-0311-1

**Published:** 2019-06-14

**Authors:** Jianxin Tan, Yajun Wang, Siliang Wang, Simeng Wu, Zhe Yuan, Xike Zhu

**Affiliations:** 1 State Key Laboratory of Reproductive Medicine, Department of Prenatal Diagnosis, Women’s Hospital of Nanjing Medical University, Nanjing Maternity and Child Health Care Hospital, Nanjing, 210004 People’s Republic of China; 2 Research Center, Shengjing Hospital of China Medical University, 36 Sanhao Street, Shenyang, 110004 People’s Republic of China; 3 Department of Medical Oncology, Shengjing Hospital of China Medical University, Shenyang, 110022 People’s Republic of China; 4 Department of Blood Transfusion, Shengjing Hospital of China Medical University, Shenyang, 110022 People’s Republic of China

**Keywords:** Thymic involution, Adipocytes, Proteomics, TGF-β1, PPARγ, Wnt/β-catenin

## Abstract

**Background:**

Adipocyte accumulation is a predominant feature of age-related thymic involution, but the mechanisms responsible for thymic adipogenesis remain to be elucidated. The aim of this study was to identify key regulators in thymic adipogenesis. We used rosiglitazone, a potent peroxisome proliferator-activated receptor γ (PPARγ) agonist, to induce adipogenic differentiation of OP9-DL1 cells, and investigated the differentially expressed proteins during adipogenic differentiation by using label-free quantitative proteomics. Furthermore, the effects of transforming growth factor β1 (TGF-β1) on rosiglitazone-induced adipogenic differentiation of OP9-DL1 cells as well as the underlying mechanisms were also investigated.

**Results:**

Proteomic analysis identified 139 proteins differed significantly in rosiglitazone-treated cells compared with dimethyl sulphoxide (DMSO)-treated cells. Rosiglitazone-induced adipogenic differentiation was inhibited by TGF-β1 treatment in OP9-DL1 cells and primary thymic stromal cells. Real-time PCR analysis showed significant increases in PPARγ and fatty acid binding protein 4 mRNA levels in rosiglitazone-treated cells, which were inhibited by TGF-β1 treatment. TGF-β1 down-regulated PPARγ expression at both mRNA and protein levels in OP9-DL1 cells. Chromatin immunoprecipitation analysis demonstrated that TGF-β1 enhanced the binding of Smad2/3 and histone deacetylase 1, but reduced the binding of H3K14ac to the promoter of PPARγ gene. TGF-β1 partially reversed the inhibitory effects of rosiglitazone on the expression of Axin2 and β-catenin protein levels. TGF-β1 inhibited rosiglitazone-induced adipogenic transformation in OP9-DL1 cells by down-regulation of PPARγ and activation of the canonical Wnt/β-catenin signaling pathway.

**Conclusion:**

Taken together, activation of TGF-β pathway may serve as a useful strategy to prevent thymic adiposity in age-related thymic involution.

**Electronic supplementary material:**

The online version of this article (10.1186/s13578-019-0311-1) contains supplementary material, which is available to authorized users.

## Introduction

The thymus represents a specialized primary lymphoid organ that provides the initial site and appropriate microenvironments to facilitate the differentiation, development and maturation of T cells [3]. After reaching its maximum weight in puberty, the thymus begins to atrophy slowly and steadily with advancing age [1]. This physiological process, known as age-related involution, is characterized by a decline in thymic weight and size, disruption of thymic architecture, loss of thymic epithelial cells, and adipocyte accumulation, thereby resulting in reduced naïve T cell output and increased susceptibility to infection, autoimmune disease, and cancer in the elderly [13]. Adipocyte accumulation is a predominant feature of age-related thymic involution and has a detrimental effect on thymic microenvironments [8, 35]. Thymic stromal cells (TSCs) can transdifferentiate into adipocytes, which represent a primary source of thymic adiposity [32, 39]. Nonetheless, the mechanisms responsible for thymic adipogenesis remain to be elucidated.

Proteomics represents a state-of-the-art, large-scale and systematic analysis, which enables identification and quantification of proteins in defined biological samples [23]. Quantitative proteomics allows the quantification of the alterations between normal and disease samples, which provides useful information to identify novel biomarkers and elucidate mechanisms in complex biological processes and conditions [5]. To the best of our knowledge, however, no studies using quantitative proteomic analysis to identify key regulators in thymic adipogenesis have been reported.

OP9-DL1 is a murine stromal cell line that mimics thymic microenvironments to support T cell development in vitro and provides a useful tool to study T lymphocyte lineage commitment [14] and thymic adipogenesis [31, 38]. In order to identify key regulators in thymic adipogenesis, the present study used rosiglitazone, a potent peroxisome proliferator-activated receptor γ (PPARγ) agonist, to induce adipogenic differentiation of OP9-DL1 cells, and investigated the differentially expressed proteins during adipogenic differentiation by using label-free quantitative proteomics. Furthermore, bioinformatic analysis showed that transforming growth factor β (TGF-β) signaling pathway is involved in adipogenic differentiation of OP9-DL1 cells. TGF-β1 is a multi-functional growth factor that has been reported to negatively regulate adipocyte differentiation in multiple cell types, such as pre-adipocyte 3T3-L1 cells [2], fibroblasts [4, 33], and mesenchymal stem cells [12]. However, it remains unknown whether TGF-β1 is involved in thymic adipogenesis, which may provide implications to age-related thymic involution. Therefore, we evaluated whether TGF-β1 inhibits rosiglitazone-induced adipogenic differentiation in OP9-DL1 cells and primary TSCs and, if so, whether the inhibitory effects of TGF-β1 are associated with down-regulation of PPARγ and activation of the canonical Wnt/β-catenin signaling pathway in OP9-DL1 cells.

## Methods

### Animals and reagents

C57BL/6 mice at ages of 6–8 weeks were procured from Nanjing Biomedical Research Institute of Nanjing University and housed under specific pathogen-free conditions in a standard controlled environment at Shengjing Hospital of China Medical University. The animal experiment protocol was reviewed and approved by the Animal Ethics Committee of Shengjing Hospital. Mouse TGF-β1 was obtained from R&D Systems (Minneapolis, MN, USA). TGF-β inhibitor LY-364947 and Wnt inhibitor IWR-1 were purchased from MedChem Express (Monmouth Junction, NJ, USA).

### Cell culture

OP9-DL1 cells were kindly provided by Dr. Yu Zhang (Peking University Health Science Center, Beijing, China) with permission from Dr. JC Zúñiga-Pflücker (University of Toronto, Toronto, Canada) and were maintained as previously described [31]. Primary TSCs were cultured from postnatal thymi by enzymatic digestion as previously described [36]. In brief, freshly dissected thymic lobes were cut into small fragments and digested for 20 min at 37 °C with RPMI-1640 medium containing 0.05% Liberase^TH^ (Roche, Basel, Switzerland) and 200 U/mL DNase I (Sangon Biotechnology, Shanghai, China). The digestion was repeated thrice. After completely dispersed, the cells were filtered through 100 μm mesh, seeded into 24-well plate at a density of 10^6^/well and cultured in DMEM/F12 medium supplemented with 3 μg/mL insulin (Biovision, Livingston, NJ, USA), 20 ng/mL epidermal growth factor (Peprotech, Rocky Hill, NJ, USA), 100 U/mL penicillin, 100 g/mL streptomycin, and 20% fetal bovine serum (FBS; Biological Industries, Kibbutz Beit-Haemek, Israel) at 37 °C in a 5% CO_2_ incubator [38]. After attachment, the thymocytes were removed by refreshing the culture medium. For induction of adipogenic differentiation, OP9-DL1 cells or TSCs were incubated with 10 μM rosiglitazone (Aladdin, Shanghai, China) for 7 days, and the medium containing rosiglitazone was refreshed every other day. Dimethyl sulphoxide (DMSO)-treated cells served as a vehicle control [31].

### Oil Red O staining

The differentiated cells were stained with Oil Red O to visualize lipid accumulation using a commercially available kit according to the manufacturer’s instructions (Leagene Biotechnology, Beijing, China) [31].

### Cell treatment and protein extraction

OP9-DL1 cells were seeded in 10 cm plates (3 × 10^5^ cells) and treated with DMSO or rosiglitazone for 7 days. After washing three times with ice-cold phosphate buffer saline, proteins were extracted by STD lysis buffer (4% SDS, 100 mM Tris-HCl, 1 mM DTT, pH 7.6), and the resultant lysis was collected with a cell scraper. The lysis was boiled at 100 °C for 15 min, and  the protein concentrations were determined by bicinchoninic acid assay (BCA).

### Trypsin digestion

The protein extract containing 200 µg of proteins from each sample was digested by the filter assisted sample preparation (FASP) procedure. Briefly, DTT (1 mol/L) was added to the sample to a final concentration of 0.1 mol/L, and the mixture was boiled at 100 °C for 5 min. After adding 200 μL of UA buffer (8 M urea and 0.15 M Tris-HCl, pH 8.0), each sample was concentrated in 30 kDa Microcon filtration devices and centrifuged at 14,000*g* for 15 min. UA buffer (200 μL) was added, and the mixture was centrifuged at 14,000*g* for 15 min. Then, the mixture was added with 200 μL of 50 mM/L iodoacetamide (IAA) in UA buffer, incubated at room temperature for 30 min, and centrifuged at 14,000*g* for 10 min. This step was repeated twice. Next, 100 μL of a 40 mM NH_4_HCO_3_ buffer was added to the mixture, and the mixture was centrifuged at 14,000*g* for 10 min. This step was repeated twice. Finally, the proteins were digested with 4 μg trypsin (in 40 mM NH_4_HCO_3_ buffer) at 37 °C for 18 h. After centrifuged at 14,000*g* for 10 min, the tryptic peptide mixtures were collected and used for liquid chromatography–mass spectrometry (LC–MS) analysis.

### LC–MS analysis

LC–MS analysis was achieved on an EASY-nLC1000 System equipped with a SC200 EASY-Column 10 cm × 150 μm column at a flow rate of 400 nL/min. The mobile phase A was 0.1% formic acid in acetonitrile (2% acetonitrile) and mobile phase B was 0.1% formic acid in acetonitrile (84% acetonitrile). The peptides were separated by the following gradient elution: 0–100 min: gradient increase from 0 to 45% for B; 100–108 min: gradient increase from 45 to 100% for B; 108–120 min: hold 100% for B. The eluted peptides were analyzed with a Q-Exactive mass spectrometer. The MS and MS/MS information was collected in the positive ion mode and acquired across the mass range of 300–1800 m/z followed by the top 20 MS/MS scans.

### Bioinformatic analysis

The raw MS data were analyzed using the MaxQuant software, and the P value of each protein was analyzed by Student’s t-test using the Perseus program. The proteins with a fold-change < 0.5 or > 2 and P < 0.05 were considered differentially expressed. The Blast2Go program was used for the functional annotations of the identified proteins and the Kyoto Encyclopaedia of Genes and Genomes (KEGG) pathway enrichment analysis.

### Real-time PCR

Total RNA was extracted from cells by using RNAiso Plus (TAKARA, Dalian, China) and  reverse-transcribed to synthesize cDNA using the PrimeScript 1st Strand cDNA Synthesis Kit (TAKARA). Specific mRNA transcripts were amplified using SYBR^®^ Premix Ex Taq™ II (TAKARA) on the 7500 Fast Real-Time PCR System (Applied Biosystems, Foster City, CA, USA) as previously described [31]. The sequences of specific primers are listed in Table 1. The relative mRNA levels were calculated from the threshold cycle (Ct) value and normalized to dehydrogenase (GAPDH).

**Table 1:** Sequences of primers used for real-time PCR

Gene symbol	Sequence (5′-3′)
PPARγ	
Forward	TTTTCCGAAGAACCATCCGATT
Reverse	ATGGCATTGTGAGACATCCCC
FABP4	
Forward	TGAAATCACCGCAGACGACA
Reverse	ACACATTCCACCACCAGCTT
β-catenin	
Forward	CCGTTCGCCTTCATTATGGA
Reverse	GGCAAGGTTTCGAATCAATCC
Fzd2	
Forward	TCATCTTTCTGTCCGGCTGCTACA
Reverse	AGCTGGCCATGCTGAAGAAGTAGA
Dvl1	
Forward	ATGAGGAGGACAATACGAGCC
Reverse	GCATTTGTGCTTCCGAACTAGC
Dvl3	
Forward	GTCACCTTGGCGGACTTTAAG
Reverse	AAGCAGGGTAGCTTGGCATTG
Axin2	
Forward	CGCCAACGACAGCGAGTT
Reverse	CGGTAAGGAGGGACTCCATCTA
CCND1	
Forward	CTCTAGTGGTCTCATGGCGT
Reverse	TTTCATCCCTACCGCTGTGT
LEF1	
Forward	CCCTACCCATCCTCACTGTC
Reverse	ATAGCTGGATGAGGGATGCC
GAPDH	
Forward	CGGTAAGGAGGGACTCCATCTA
Reverse	TTGCTGTTGAAGTCGCAGGAG

### Western blot analysis

Whole cell lysates were prepared in ice-cold radioimmunoprecipitation (RIPA) lysis buffer (Beyotime Institute of Biotechnology, Shanghai, China), resolved on sodium dodecyl sulfate polyacrylamide gel electrophoresis (SDS-PAGE) and transferred electrophoretically onto polyvinylidene difluoride (PVDF) membranes (Millipore, Bedford, MA, USA) as described previously [36]. After blocking with 5% (w/v) skim milk, the membranes were probed overnight at 4 °C with rabbit polyclonal antibodies against β-catenin and PPARγ (Wanlei, Shenyang, China) and a rabbit monoclonal antibody against Axin2 (Abcam, Cambridge, MA, USA), followed by incubated with appropriate horseradish peroxidase (HRP)-conjugated secondary antibodies. Subsequently, specifically binding of the primary antibodies was detected by the enhanced chemiluminescence (ECL) detection system. β-Actin was detected in parallel as an internal control for normalization.

### Immunofluorescence

OP9-DL1 cells were cultured on coverslips in 6-well plates, fixed in 4% paraformaldehyde at room temperature for 1 h, and permeabilized in 0.5% Triton X-100 for 20 min. After blocked with 5% FBS for 20 min at room temperature, the coverslips were incubated overnight at 4 °C with a rabbit monoclonal antibody against β-catenin (1:100 diluted, Abcam). Subsequently, the coverslips were reacted with Cy3-labeled anti-rabbit IgG antibody, followed by stained the cell nuclei with 4′, 6-diamidino-2-phenylindole (DAPI). Finally, the coverslips were detected and photographed under a fluorescence microscopy. Quantification of the nuclear/cytoplasmic β-catenin ratio was performed using ImageJ software as described previously [34]. Briefly, the images were converted into 8-bit scale images, and the nuclear/cytoplasmic β-catenin ratio was determined by measuring the density of a randomly selected region in the nucleus and cytoplasm.

### Chromatin immunoprecipitation (ChIP) assay

Chromatin immunoprecipitation assay was performed to evaluate the binding of Smad2/3, HDAC1 and H3K14ac to the PPARγ gene promoter by using a commercially available kit (Active Motif, Carlsbad, CA, USA) following the manufacturer’s instructions. In brief, OP9-DL1 cells were treated with TGF-β1 and cross-linked with 1% formaldehyde for 10 min at 37  °C, followed by nuclei extraction and enzyme digestion of chromatin. The sheared chromatin was pre-cleared and incubated at 4 °C overnight with 2 μg of rabbit polyclonal antibodies against Smad2/3 (R&D Systems) and H3K14ac (Active Motif) and a mouse monoclonal antibody against HDAC1 (Active Motif). The DNA–protein complexes were captured using protein G magnetic beads, and the DNA–protein cross-links were reversed at 95 °C for 15 min. After sequentially treated with  proteinase K and RNase A, the DNA samples were used as the template for PCR amplification. The primers used for PPARγ promoter (− 74 to − 146 bp) are: forward, 5′-ACATCGGTCTGAGGGACACGGG-3′ and 5′-TACCTGGCCGCCTTGCTCCT-3′). IgG was used as a negative control to confirm the specificity, and precleared chromatin was designated as input control. PCR reaction products were electrophoresed on 1.5% agarose gels with ethidium bromide.

### Statistical analysis

Data are presented as mean ± standard deviation (SD). Differences among groups were evaluated by one-way analysis of variance, and the Bonferroni post hoc test was used for post hoc comparisons. Graphs were constructed and statistical analysis was carried out using GraphPad Prism 5 (GraphPad Software, La Jolla, CA, USA). A P value less than 0.05 was considered to be statistically significant.

## Results

### Identification of differentially expressed proteins during adipogenic transformation in OP9-DL1 cells

**Fig. 1:**
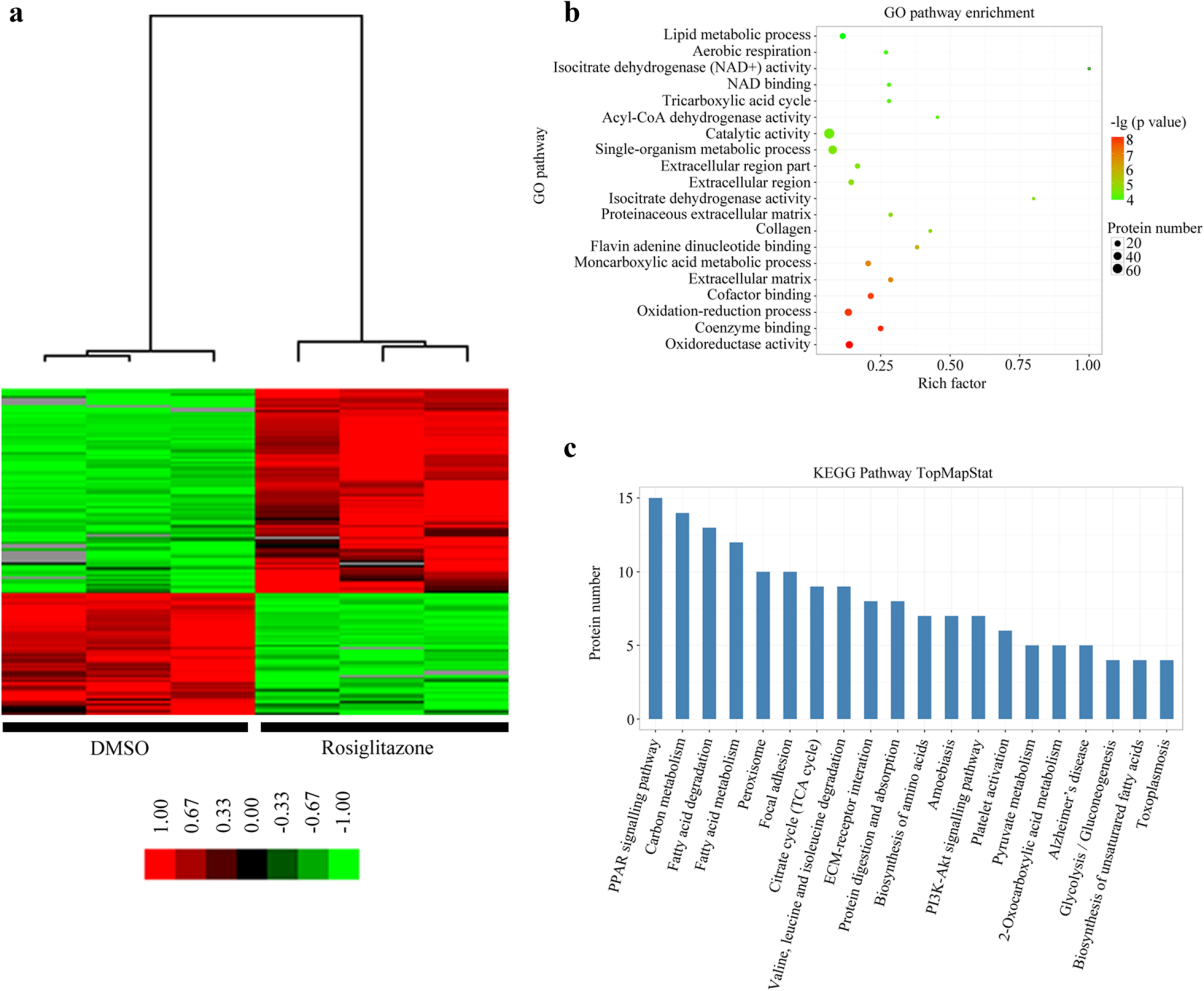
Proteomic analysis of proteins differed significantly in rosiglitazone-treated cells compared with dimethyl sulphoxide (DMSO)-treated cells. **a** Heatmap of significantly altered proteins between rosiglitazone- and DMSO-treated OP9-DL1 cells (n = 3). GO enrichment analysis (**b**) and KEGG pathway analysis (**c**) of all differentially expressed proteins due to rosiglitazone treatment. *Rosi* rosiglitazone

To investigate the mechanisms responsible for thymic adiposity, proteomic analysis was conducted on rosiglitazone- and DMSO-treated OP9-DL1 cells. As a result, we identified 21,195 unique peptides matching to 3062 unique proteins in OP9-DL1 cells by using LC–MS analysis. Unpaired t-test analysis showed that 139 proteins differed significantly at P < 0.05 with a fold change > 2. Among them, 87 proteins were up-regulated and 52 proteins were down-regulated in rosiglitazone-treated cells compared with DMSO-treated cells. The data on the top 10 proteins up-regulated and 10 proteins down-regulated are listed in Table 2. Unsupervised hierarchical clustering analysis of differentially expressed proteins revealed that DMSO-treated cells are distinctly different from rosiglitazone-treated cells (Fig. 1a). In order to identify the potential functions of these differentially expressed proteins, we performed GO pathway enrichment analysis with Blast2Go program, showing that the GO pathways of most differentially expressed proteins were enriched in lipid metabolic process, followed by aerobic respiration and isocitrate dehydrogenase (NAD+) activity (Fig. 1b). Furthermore, the differentially expressed proteins were classified into 161 pathways by using the KEGG pathway database, the top 20 of which are shown in Fig. 1c. The PPARγ was the top pathway, followed by the carbon metabolism pathway. Interestingly, we noted that the TGF-β signaling pathway was also significantly enriched (Additional file 1: Table S1), with down-regulation of some member proteins. Earlier evidence indicates that the TGF-β signaling pathway inhibits adipogenesis by suppressing the expression of PPARγ and C/EBPs [12]. Therefore, we hypothesized that TGF-β1 inhibits the transdifferentiation of TSCs to adipocytes in the thymus [32].

**Table 2:** Top 20 proteins found to be significantly altered in rosiglitazone-treated cells compared with DMSO-treated cells

Protein names	Peptides	Sequence coverage (%)	Molecular weight (kDa)	Fold changes	P values
Acsl1	33	55.8	78.0	149.3	1.1E−04
CD36	12	32.4	52.7	64.4	0.037
FABP4	8	56.1	14.7	39.9	2.8E−04
Pcx	33	36.4	129.8	18.7	5.1E−06
Lpl	12	32.5	53.1	9.6	0.003
Col15a1	12	10.9	140.5	8.0	6.4E−05
Acox1	13	30.4	74.6	7.9	6.5E−06
FABP5	7	57	15.1	7.7	0.015
Aldh6a1	9	26.2	57.9	6.7	7.2E−05
Mgst1	3	17.9	25.8	6.3	0.002
Emilin1	21	34.9	107.6	0.06	4.6E−05
Abcb1b	1	8.9	141.0	0.09	8.7E−04
Dpysl3	17	45.1	73.9	0.11	5.8E−05
IL1RN	7	70.4	18.0	0.11	2.1E−04
Fibronectin	3	49.3	262.8	0.18	5.8E−05
Col12a1	14	7.3	340.2	0.20	0.020
Wls	4	7	62.2	0.20	1.2E−04
Cnn3	10	45.5	36.4	0.23	3.5E−05
Lmcd1	10	34.8	41.0	0.25	0.002
Rai14	11	16.2	108.9	0.25	0.007

### TGF-β1 attenuated rosiglitazone-induced adipogenic differentiation in OP9-DL1 cells

**Fig. 2:**
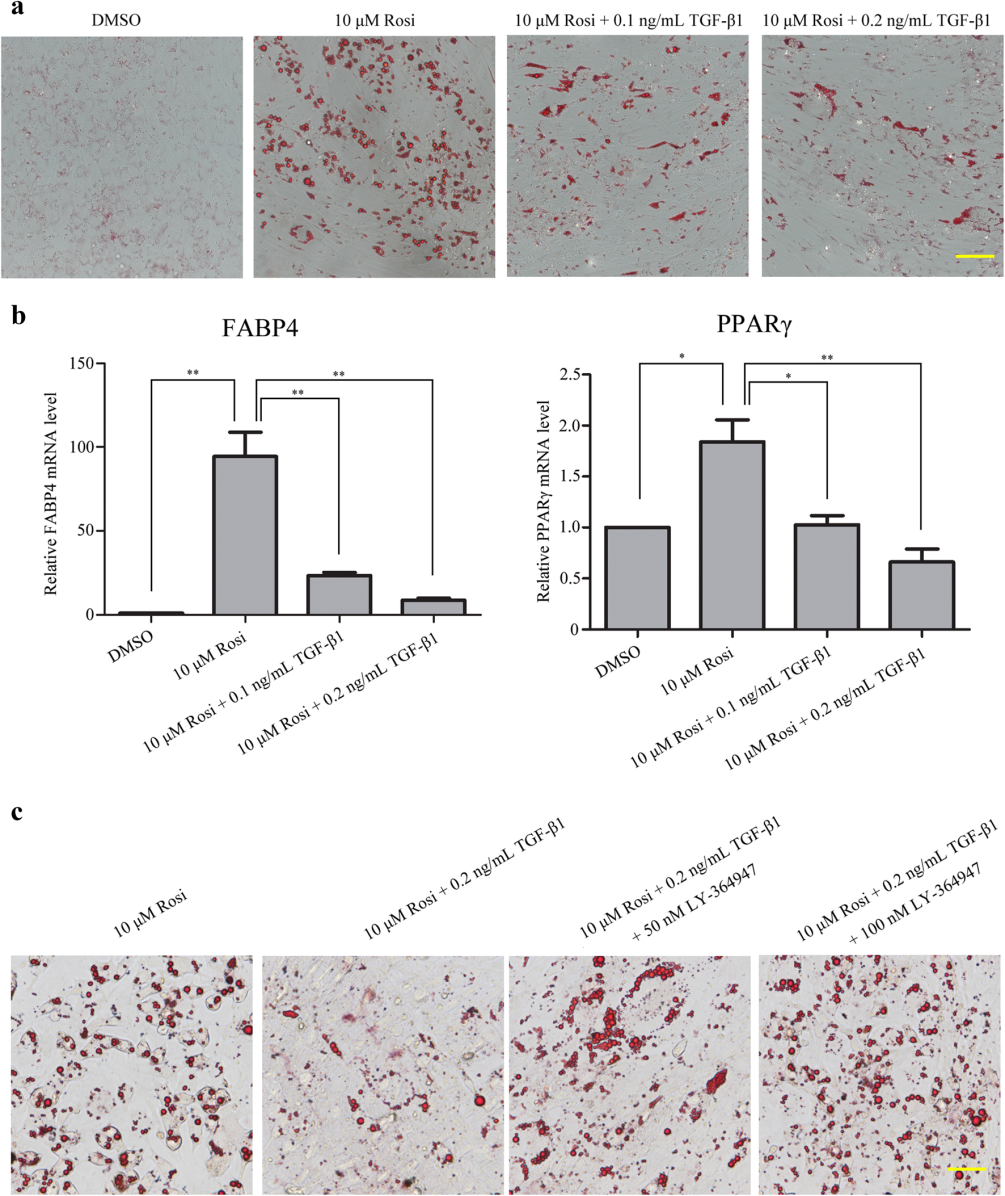
TGF-β1 attenuated rosiglitazone-induced adipogenic differentiation in OP9-DL1 cells. OP9-DL1 cells were treated with various concentrations of TGF-β1 for 7 days in the presence of rosiglitazone. **a** Lipid accumulation was visualized by Oil Red O staining, and representative photographs of accumulated lipids are shown. **b** Real-time PCR was performed to detect FABP4 and PPARγ mRNA levels. The data were normalized to GAPDH, and the graph represents the results of three independent experiments. **P < 0.01; *P < 0.05. **c** OP9-DL1 cells were treated with rosiglitazone and TGF-β1 in the presence of LY-364947 for 7 days. Representative images of accumulated lipids in differentiating OP9-DL1 cells visualized by Oil Red O staining are presented. Bars, 50 μM. *Rosi* rosiglitazone

To test our hypothesis, OP9-DL1 cells were treated with various concentrations of TGF-β1 in the presence of rosiglitazone. After 7 days treatment, lipid accumulation was determined by Oil Red O staining. As shown in Fig. 2a, TGF-β1 reduced lipid accumulation compared with differentiated OP9-DL1 cells in a dose-dependent fashion. Additionally, we further detected the mRNA levels of PPARγ and fatty acid-binding protein 4 (FABP4) to characterize the effects of TGF-β1 on OP9-DL1 adipocyte differentiation. Using real-time PCR analysis, we found that the mRNA levels of PPARγ and FABP4 were dramatically increased in rosiglitazone-treated cells compared with DMSO-treated cells. However, TGF-β1 treatment dose-dependently attenuated the increases in PPARγ and FABP4 expression induced by rosiglitazone in OP9-DL1 cells (Fig. 2b). Furthermore, the effects of TGF-β inhibitor LY-364947 on lipid accumulation in OP9-DL1 cells were also evaluated by Oil Red O staining. As shown in Fig. 2c, LY-364947 obviously reversed the inhibitory effects of TGF-β1 on OP9-DL1 adipocyte differentiation. Taken together, our data suggest that TGF-β1 specifically inhibits rosiglitazone-induced adipogenic differentiation in OP9-DL1 cells.

### TGF-β1 down-regulated PPARγ expression in OP9-DL1 cells

**Fig. 3:**
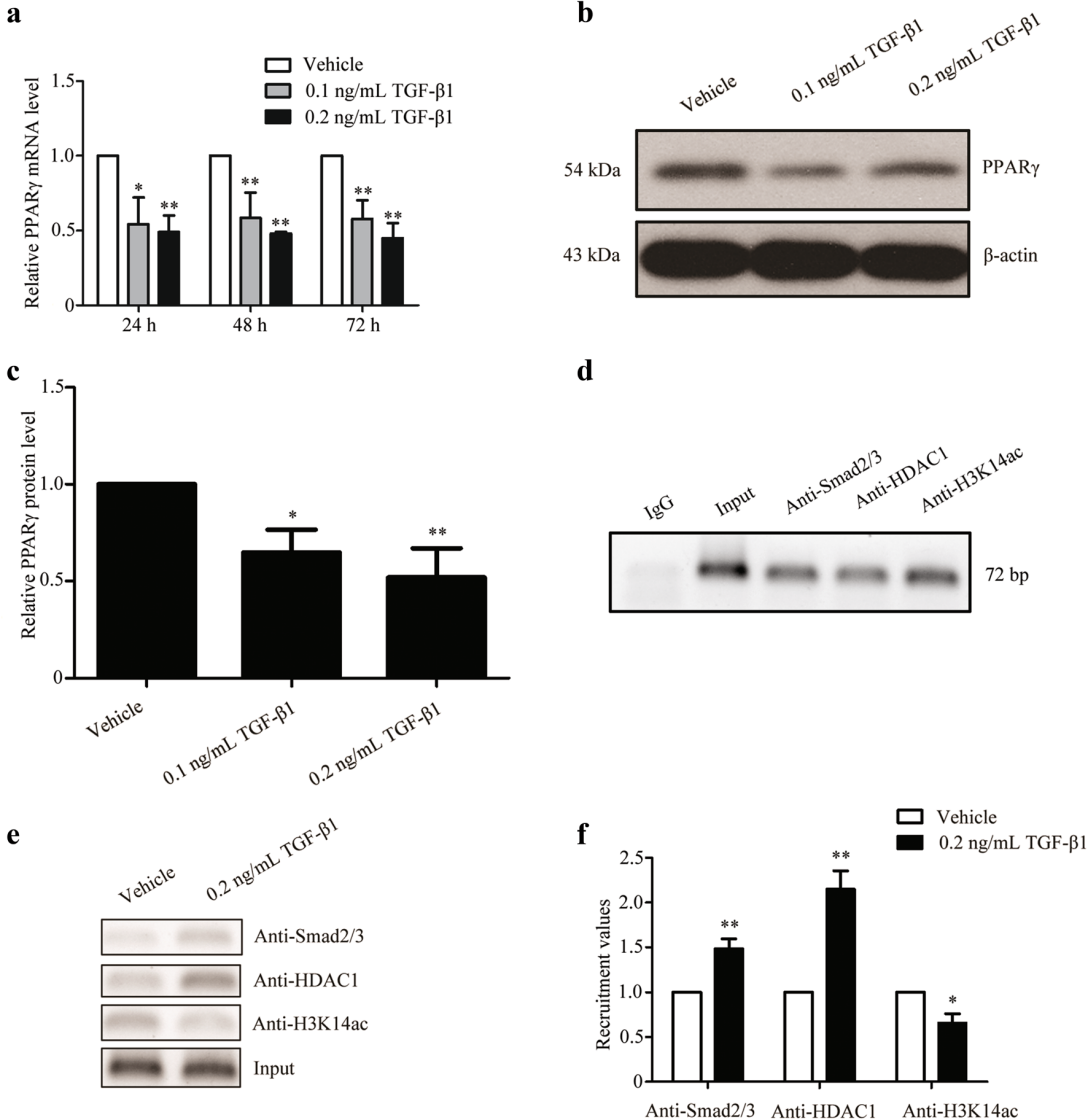
TGF-β1 down-regulated PPARγ expression level in OP9-DL1 cells. **a** OP9-DL1 cells were treated with various concentrations of TGF-β1 for different intervals, and PPARγ mRNA levels were examined by real-time PCR. GAPDH served as an internal control. **b** After treatment with TGF-β1 for 72 h, total protein was extracted and PPARγ protein levels were determined by Western blot analysis. Representative blots are shown, and protein size is expressed as kDa. **c** Quantitative densitometric analysis was performed by using β-actin as a loading control. Quantification data represent results obtained from three independent experiments. ChIP assay was performed in OP9-DL1 cells without treatment (**d**) and treated with 0.2 ng/mL TGF-β1 or vehicle for 6 h (**e**). Chromatin was immunoprecipitated with antibodies against Smad 2/3, HDAC1 and H3K14ac, and the PPARγ promoter fragment was amplified by PCR. Densitometric analysis was conducted in comparison to vehicle control. Data are presented as averages from three independent experiments (**f**). **P < 0.01; *P < 0.05 vs vehicle

PPARγ is known to be a key adipogenic transcription factor. Next, we investigated the effects of TGF-β1 on PPARγ expression in OP9-DL1 cells. Real-time PCR analysis showed that TGF-β1 treatment remarkably reduced PPARγ mRNA levels in OP9-DL1 cells in a dose-dependent and time-dependent manner (Fig. 3a). As shown in Fig. 3b, c, the observations in Western blot analysis were consistent with the results in real-time RT-PCR analysis. Furthermore, a ChIP assay was performed in OP9-DL1 cells exposed or not exposed to TGF-β1. After treatment with 0.2 ng/mL TGF-β1, nuclear protein–DNA complexes were prepared and subjected to ChIP assay by using a specific antibody against Smad2/3. The results showed that Smad2/3 directly bound to the promoter of PPARγ in the absence of TGF-β1 (Fig. 3d), and TGF-β1 treatment increased the recruitment of Smad2/3 to the promoter of PPARγ (Fig. 3e, f). In general, histone acetylation is closely associated with transcriptional activation. Protein-chromatin complexes were immunoprecipitated with antibodies against HDAC1, a transcriptional suppressor, and acetylated histone (H3K14ac), an indicator of active transcription. As shown in Fig. 3e, f, TGF-β1 treatment enhanced the recruitment of HDAC1 and reduced the level of H3K14ac at the promoter region of the PPARγ gene. These observations demonstrate that TGF-β1 transcriptionally down-regulates PPARγ in OP9-DL1 cells.

### TGF-β1 inhibited rosiglitazone-induced adipogenic transformation in OP9-DL1 cells by activating the canonical Wnt/β-catenin signaling pathway

**Fig. 4:**
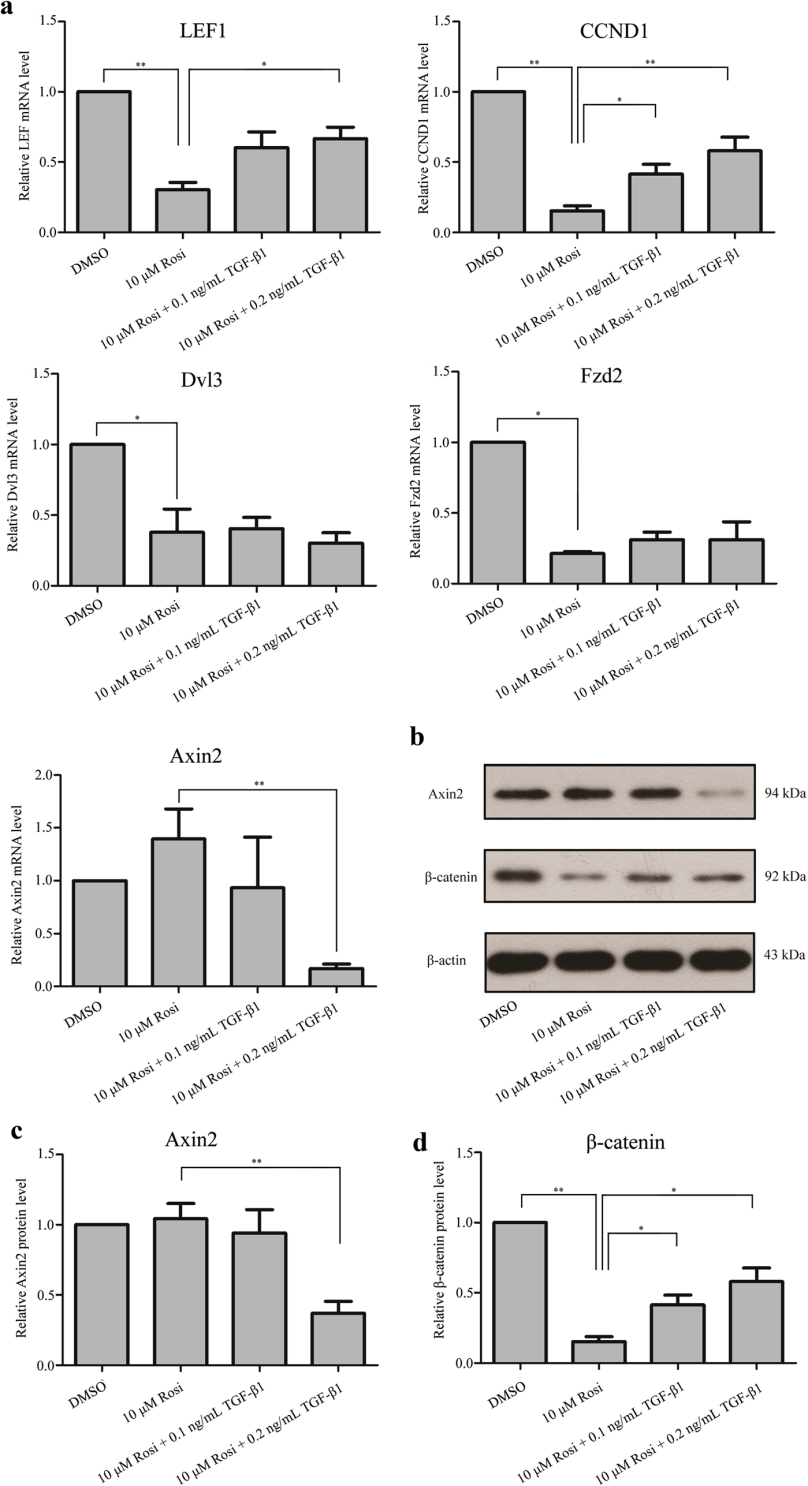
TGF-β1 activated Wnt/β-catenin signaling pathway in OP9-DL1 cells. OP9-DL1 cells were treated with various concentrations of TGF-β1 for 7 days in the presence of rosiglitazone. **a** LEF1, CCND1, Dvl3, Fzd2 and Axin2 mRNA levels were measured by real-time PCR and normalized to GAPDH. **b** β-catenin and Axin2 protein levels were assessed by Western blot analysis. The blots shown are representative of three independent experiments. **c**, **d** Columns represent mean ± SD from three independent experiments. **P < 0.01; *P < 0.05. *Rosi* rosiglitazone

**Fig. 5:**
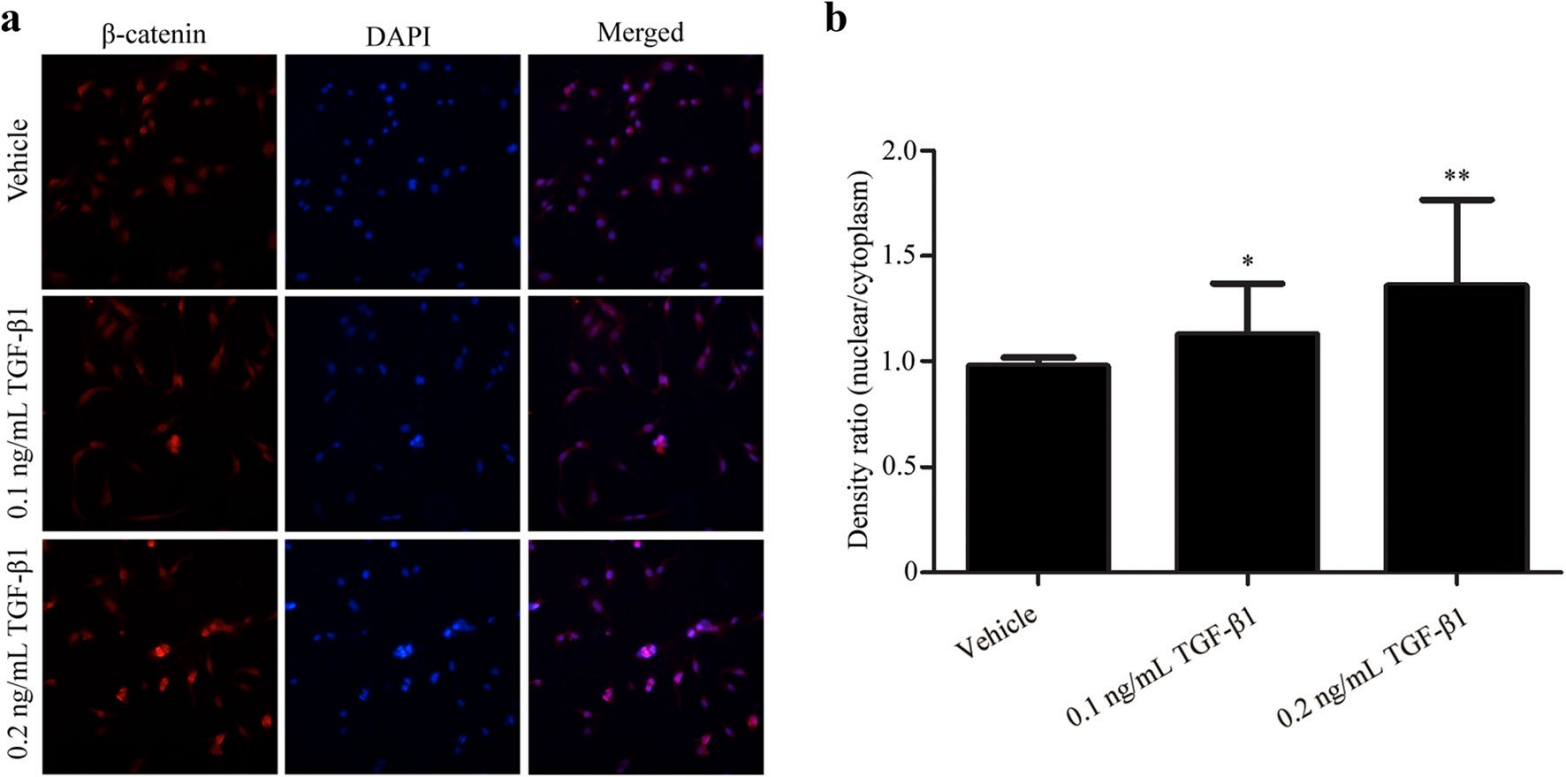
TGF-β1 promoted nuclear translocation of β-catenin in OP9-DL1 cells. **a** Cells were treated with various concentrations of TGF-β1 for 72 h. Immunofluorescence staining was imaged using anti-β-catenin (red), and the nuclei were counterstained with DAPI (blue). Magnification, ×200. **b** The nuclear/cytoplasmic β-catenin ratio was quantified using ImageJ software and determined by measuring the density of a randomly selected region in the nucleus and cytoplasm. Cells were counted from three different microscopic fields, and results are expressed as mean ± SD. **P < 0.01; *P < 0.05 vs vehicle

**Fig. 6:**
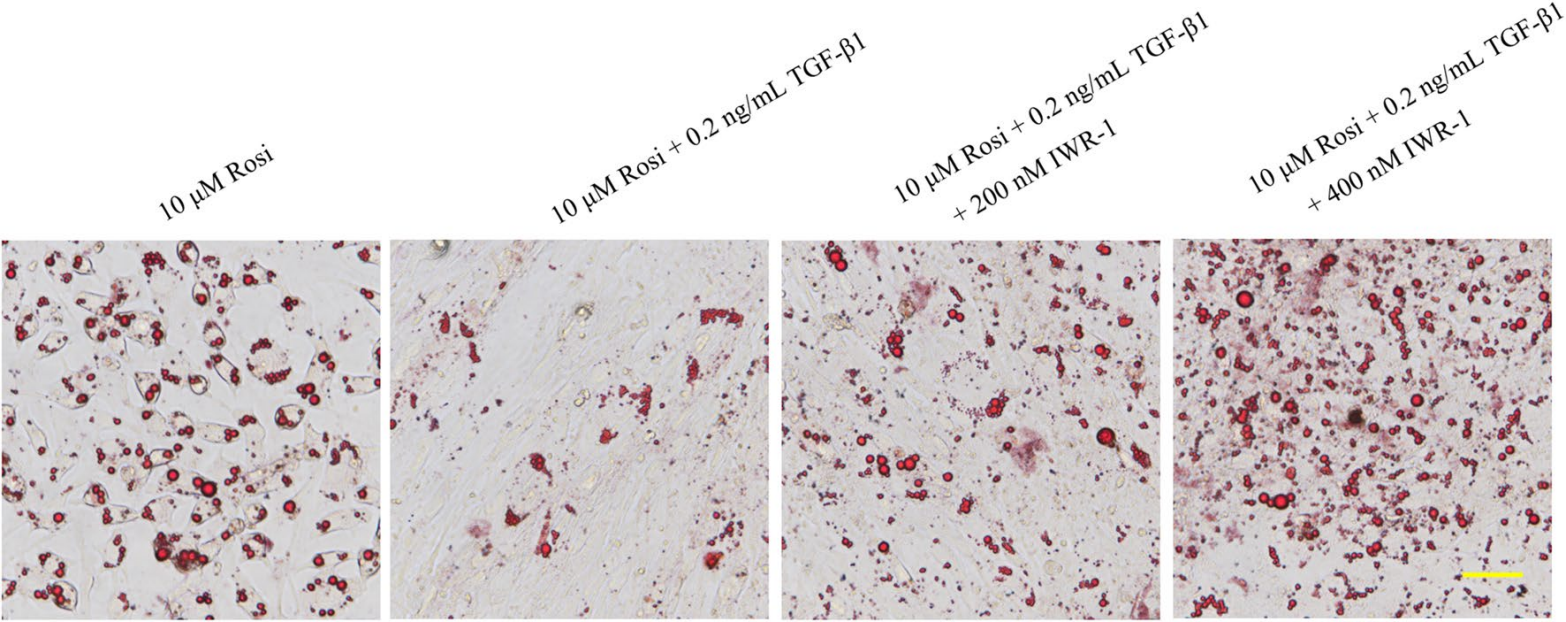
IWR-1, a small molecule inhibitor of the Wnt/β-catenin pathway, restored the inhibitory effects of TGF-β1 on OP9-DL1 adipocyte differentiation. OP9-DL1 cells were treated with rosiglitazone and TGF-β1 in the presence of IWR-1 for 7 days, and accumulated lipids were visualized by Oil Red O staining. Bars, 50 μM. *Rosi* rosiglitazone

**Fig. 7:**
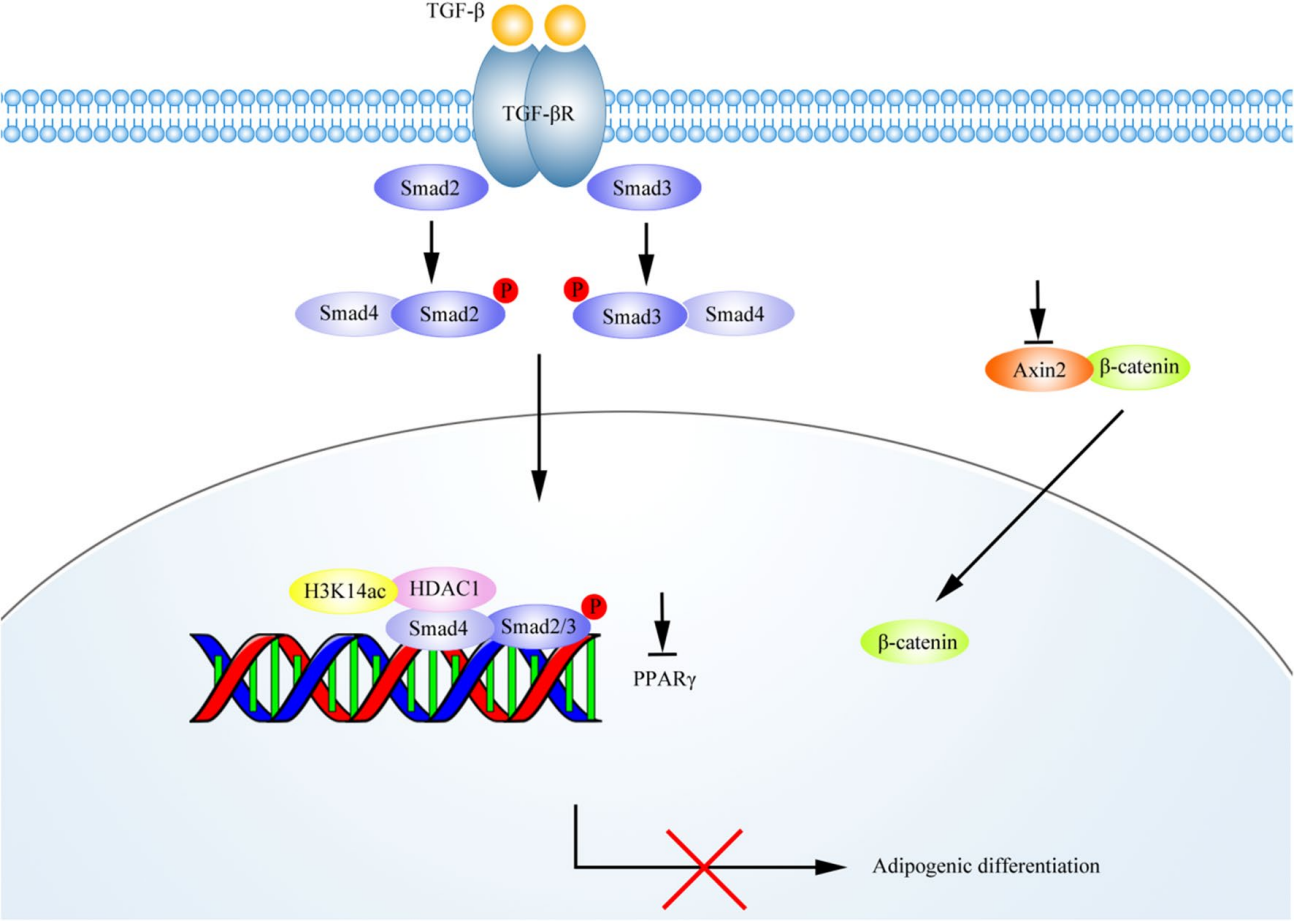
Schematic diagram of the mechanisms by which TGF-β1 attenuates rosiglitazone-induced adipogenic differentiation in OP9-DL1 cells. TGF-β1 increases the recruitment of Smad2/3 and HDAC1 and reduces the level of H3K14ac at the promoter region of the PPARγ gene, thereby transcriptionally down-regulates PPARγ in OP-DL1 cells. TGF-β1 down-regulates Axin2 expression to induce nuclear translocation of β-catenin in OP9-DL1 cells

The canonical Wnt/β-catenin signaling pathway has been reported to negatively regulate adipocyte differentiation [6]. To assess whether the Wnt/β-catenin signaling pathway is involved in TGF-β1-mediated inhibition on adipocyte differentiation, the expression levels of several members in the Wnt/β-catenin pathway were detected by real-time PCR. As illustrated in Fig. 4a, the mRNA levels of lymphoid enhancer binding factor 1 (LEF1), cyclin D1 (CCND1), Dvl3 and Fzd2 were significantly reduced in rosiglitazone-treated cells, suggesting inactivation of the Wnt/β-catenin pathway during adipocyte differentiation in OP9-DL1 cells. However, the mRNA levels of β-catenin, Dvl1 and Axin2 were not altered (data for β-catenin and Dvl1 are not shown). More importantly, TGF-β1 treatment remarkably restored the decreases in LEF1 and CCND1 mRNA levels induced by rosiglitazone (Fig. 4a). β-catenin is a key player in the Wnt signaling pathway and has been reported to be regulated by post-transcriptional mechanisms. Therefore, Western blot analysis was conducted to evaluate the protein levels of β-catenin in OP9-DL1 cells. We found that rosiglitazone treatment resulted in an obvious decrease in β-catenin protein level, which was significantly attenuated by TGF-β1 treatment (Fig. 4b, d). Notably, TGF-β1 treatment, particularly at high concentration, remarkably reduced the expression of Axin2 at both mRNA (Fig. 4a) and protein levels (Fig. 4b, c). In the absence of Wnt, β-catenin is sequestered in the cytoplasm. Upon activation, cytoplasmic β-catenin dissociates from inhibitory complexes, accumulates in the cytoplasm, and translocates into the nucleus [22]. Next, the subcellular localization of β-catenin was analyzed by immunofluorescence analysis. As shown in Fig. 5, TGF-β1 treatment induced nuclear translocation of β-catenin in OP9-DL1 cells, suggesting activation of the Wnt/β-catenin pathway. Additionally, Oil Red O staining showed that IWR-1, a small molecule inhibitor of the Wnt/β-catenin pathway, restored the inhibitory effects of TGF-β1 on OP9-DL1 adipocyte differentiation (Fig. 6). These data suggest that TGF-β1 inhibited rosiglitazone-induced adipogenic transformation in OP9-DL1 cells by down-regulation of PPARγ and activation of the canonical Wnt/β-catenin signaling pathway (Fig. 7).

### TGF-β1 suppressed rosiglitazone-induced adipogenic transformation in primary TSCs

**Fig. 8:**
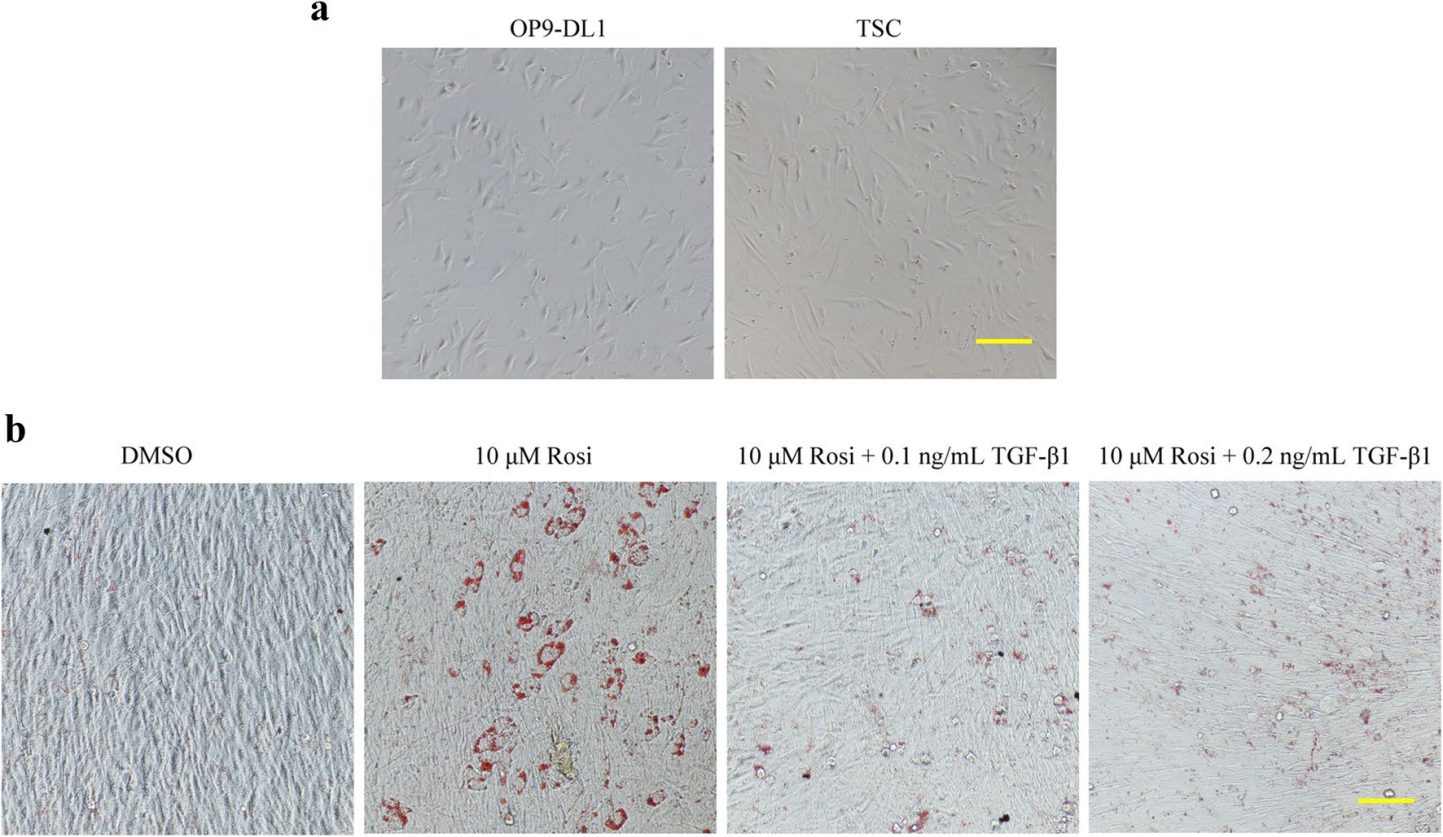
TGF-β1 suppressed rosiglitazone-induced adipogenic transformation in primary TSCs. **a** The morphology of TSCs and OP9-DL1 cells observed by optical microscope. **b** TSCs were treated with various concentrations of TGF-β1 for 7 days in the presence of rosiglitazone, and Oil Red O staining was carried out to visualize accumulated lipids. Bars, 50 μM. *Rosi* rosiglitazone

To further determine the effects of TGF-β1 on adipocyte differentiation in thymic cells, primary TSCs were cultured as previously described. The morphology of TSCs was comparable to that of OP9-DL1 cells (Fig. 8a). Similarly, Oil Red O staining also showed that TGF-β1 suppressed rosiglitazone-induced adipogenic transformation in primary TSCs (Fig. 8b).

## Discussion

In the present study, we performed a comprehensive analysis of differentially expressed proteins in OP9-DL1 cells by using label-free quantitative proteomics, which identified 87 proteins up-regulated and 52 proteins down-regulated in rosiglitazone-treated cells compared with DMSO-treated cells. KEGG pathway analysis revealed that PPARγ, carbon metabolism, TGF-β signaling pathway were significantly enriched. Our further experiments showed that TGF-β1 suppressed rosiglitazone-induced adipogenic differentiation in OP9-DL1 cells and primary TSCs, and the inhibitory effects of TGF-β1 are associated with down-regulation of PPARγ and activation of the Wnt/β-catenin signaling pathway in OP9-DL1 cells. Taken together, our results demonstrated that TGF-β1 is a key player in thymic adipogenesis and may represent a promising target to prevent thymic adiposity in age-related thymic involution.

Rosiglitazone, a PPARγ ligand, is used for treatment of type 2 diabetes primarily due to its insulin-sensitizing property [17, 24]. Because PPARγ is a master regulator of adipocyte differentiation, an increase in adiposity and weight gain are commonly observed side-effects in rosiglitazone treatment [29]. Furthermore, rosiglitazone has also been used to induce transdifferentiation of multipotential cells into adipocytes, such as proadipocytes [16], bone marrow-derived mesenchymal stem cells [19], and embryonic stem cells [37]. Yang and colleagues [38] have reported that OP9-DL1 cells acquire adipocyte morphology upon rosiglitazone treatment. In the present study, we also successfully induced the adipogenic transformation of OP9-DL1 cells into adipocytes by using rosiglitazone. Furthermore, our label-free quantitative proteomics analysis identified a total of 139 proteins differentially expressed in rosiglitazone-treated cells compared with DMSO-treated cells, among which hepatic long-chain acyl-CoA synthetase 1 (Acsl1), CD36 and FABP4 showed the highest fold-changes. Acsl1 belongs to a family of long-chain acyl-CoA synthetase that catalyzes the synthesis of acyl-CoA from long-chain fatty acids [30]. Consistent with our results, Dunning and colleagues [7] found that rosiglitazone increases Acsl1 levels in cumulus–oocyte complexes. CD36 is a transmembrane glycoprotein involved in the cellular uptake of long-chain fatty acids [10]. FABP4, an intracellular fatty acid binding protein, is primarily expressed in macrophages and adipocytes and regulates fatty acid storage and lipolysis [9]. More importantly, CD36 and FABP4 are believed to be target genes of PPARγ. Previous studies support our findings by showing that rosiglitazone induces the expression of CD36 and FABP4 [15, 21]. However, further investigations are needed to uncover the functions of other altered proteins in adipogenic transformation of OP9-DL1 cells.

Despite up-regulation in adipocytes of obese mouse, TGF-β1 is known to be a potent negative regulator of adipocyte differentiation [2, 27]. TGF-β1 inhibits adipocyte differentiation by Smad3-mediated inhibition of C/EBP and down-regulation of PPARγ via the β-catenin pathway [4, 26]. In this study, we consistently found that TGF-β1 treatment dramatically inhibited rosiglitazone-induced adipocyte differentiation and PPARγ and FABP4 up-regulation in OP9-DL1 cells. Additionally, real-time PCR and Western blot analysis showed that TGF-β1 treatment obviously down-regulated PPARγ mRNA and protein levels, respectively. Acetylated histones at promoters are mechanistically linked to genes with active transcription, and Smad proteins binding to the promoter region of target genes are required for the TGF-β-mediated transcriptional activity [20]. Therefore, ChIP assay was performed to evaluate the binding of Smad2/3, HDAC1 and H3K14ac to the promoter of PPARγ upon treatment with TGF-β1. Our data found that Smad2/3, HDAC1 and H3K14ac constitutively bound to the PPARγ promoter, and increased binding of Smad2/3 and HDAC1 and reduced H3K14ac binding were observed in response to TGF-β1. Our observations are consistent with those by Gong et al. [11] in mouse cardiac fibroblasts. However, the precise region of the PPARγ promoter by which TGF-β1 mediates the inhibition of PPARγ gene expression is required to be further clarified.

β-Catenin is a nucleocytoplasmic shuttling protein and a central component and mediator of the Wnt signaling pathway [25]. In the absence of Wnt, cytoplasmic β-catenin protein is constantly destructed by a multi-protein complex, which is consisted of the scaffolding protein Axin, the adenomatous polyposis coli (APC) tumor suppressor protein, and glycogen synthase kinase 3 (GSK3). When activation, β-catenin dissociates from the complex, translocates into the nucleus, and thereby regulates its target genes [22]. Earlier evidence indicates a link between TGF-β and Wnt signaling [28]. In human bone marrow stromal cells, TGF-β up-regulates Wnt ligand to activate Wnt/β-catenin signaling, inhibits adipocyte differentiation, and promotes chondrocyte differentiation [40]. In contrast, TGF-β prevents adipocyte differentiation of 3T3-L1 pre-adipocyte through Wnt/β-catenin-independently mechanisms [18, 33]. In the current study, we found that the mRNA levels of LEF1, CCND1, Dvl3 and Fzd2 were significantly decreased in rosiglitazone-treated cells, suggesting a negative role of the Wnt/β-catenin pathway in adipocyte differentiation of OP9-DL1 cells. More importantly, TGF-β1 down-regulated the mRNA and protein levels of Axin2 and facilitated the translocation of β-catenin into the nucleus. Meanwhile, IWR-1, a small molecule inhibitor of the Wnt/β-catenin pathway, partially reversed the inhibitory effects of TGF-β1 on OP9-DL1 adipocyte differentiation. Altogether, our findings suggest that TGF-β1 specifically activates the Wnt/β-catenin pathway to inhibit OP9-DL1 adipocyte differentiation. Nevertheless, the targets by which TGF-β1 activates the Wnt/β-catenin pathway are needed to be further elucidated. Furthermore, whether TGF-β1 treatment reverses age-related thymic involution in aged mice also warrants further investigations.

## Conclusions

In summary, our proteomic analysis identified a number of differentially expressed proteins and associated signaling pathways in the adipocyte differentiation of OP9-DL1 cells. Moreover, we demonstrated that TGF-β1 inhibits rosiglitazone-induced adipogenic differentiation in OP9-DL1 cells and primary TSCs, and the inhibitory effects of TGF-β1 are associated with down-regulation of PPARγ and activation of the canonical Wnt/β-catenin signaling pathway in OP9-DL1 cells. Our observations not only provide the basic information for identification of key regulator in thymic adipogenesis, but also reveal a novel role of TGF-β1 in thymic adiposity. Therefore, activation of TGF-β pathway may serve as a useful strategy to prevent thymic adiposity in age-related thymic involution.

## Additional file

**Additional file 1: Table S1.** The complete list of 161 pathways from enrichment analysis of differentially expressed proteins according to KEGG database.

## Data Availability

The full list of the differentially expressed proteins identified by proteomics will be available from the corresponding author upon request.
